# Preparatory behaviours and condom use during receptive and insertive anal sex among male-to-female transgenders (*Waria*) in Jakarta, Indonesia

**DOI:** 10.7448/IAS.17.1.19343

**Published:** 2014-12-19

**Authors:** Ciptasari Prabawanti, Arie Dijkstra, Pandu Riono, Gagan Hartana Tb

**Affiliations:** 1 Social Psychology Department, Faculty of Behavioral and Social Sciences, University of Groningen, Groningen, The Netherlands; 2 Department of Population Studies and Biostatistic, Faculty of Public Health, University of Indonesia, Depok, Indonesia; 3 Faculty of Psychology, University of Indonesia, Depok, Indonesia

**Keywords:** transgender, theory of planned behaviour, preparatory behaviours, condom use, HIV/AIDS, Indonesia

## Abstract

**Introduction:**

The male-to-female transgender (*waria*) is part of a key population at higher risk for HIV. This study aims to test whether psychosocial determinants as defined by the theory of planned behaviour (TPB) can explain behaviours related to condom use among *waria*. Three preparatory behaviours (getting, carrying, and offering a condom) and two condom use behaviours (during receptive and insertive anal sex) were assessed.

**Methods:**

The study involved 209 *waria*, recruited from five districts in Jakarta and interviewed by using structured questionnaires. Specific measures were developed to study attitudes, subjective norms and perceived behavioural control (PBC) in order to predict intentions and behaviours.

**Results:**

The explained variance between intentions with regard to three preparatory behaviours and two condom uses ranged between 30 and 57%, and the variance between the actual preparatory behaviours of three preparatory and two condom uses ranged between 21 and 42%. In our study, as with several previous studies of the TPB on HIV protection behaviours, the TPB variables differed in their predictive power. With regard to intention, attitude and PBC were consistently significant predictors; attitude was the strongest predictor of intention for all three preparatory behaviours, and PBC was the strongest predictor of intention for condom use, both during receptive and insertive anal sex. TPB variables were also significantly related to the second parameter of future behaviour: actual (past) behaviour. TPB variables were differentially related to the five behaviours. Attitude was predictive in three behaviours, PBC in three behaviours and subjective norms in two behaviours.

**Conclusions:**

Our results have implications for the development of interventions to target preparatory behaviours and condom use behaviours. Five behaviours and three psychological factors as defined in the TPB are to be targeted.

## Introduction

### *Waria* and sexual risk behaviour

*Waria* is an Indonesian term for the male-to-female transgender. Because *waria* frequently practice anal sex in commercial and non-commercial settings, they are therefore identified as part of a key population at higher risk for HIV and other STIs (sexually transmitted infections). *Waria* are either (1) people who appear androgynous or behave androgynously or (2) biological males who cross-dress and/or adopt the behavioural and societal roles of females. The first definition of *waria* refers to androgynous gender behaviour and the second definition refers to a socially constructed identity [1]. In short, *waria* refers to individuals whose gender expression and identity are in contrast with their biological sex.

Although the latest 2011 data showed that condom use among *waria* in Jakarta had increased to 59% (as compared to 15% in 2007) [2,3], the prevalence of HIV in Jakarta is still high; above 30% [2,3]. Thus, *waria* condom use seems still insufficient to decrease HIV transmission in this population. Indeed, several studies have estimated that a consistent condom use of 70–80% is needed during anal sex among male same-sex partners in order for it to be effective [4]. Therefore, it is important to further develop effective interventions that aim to increase condom use. Health promotion interventions can target the psychosocial determinants of condom use. However, at present very little is known about these psychosocial determinants in *waria*. This study aims to fill this gap by examining these psychosocial factors in *waria*.

In the present study, we examined condom use behaviours on the basis of the theory of planned behaviour (TPB). Given that this model has predicted health-related behaviours [5], including condom use behaviours [6–9], we also expected it to help explain *waria* condom use during anal sex behaviours. The TPB postulates three conceptually independent determinants of intention. The first is the attitude towards the behaviour itself, whether favourable or unfavourable. The second predictor is subjective norms, the perceived social pressure to perform or not to perform the behaviour. The third predictor of intention is the degree of perceived behavioural control (PBC): the extent to which people feel that they have control over performing the behaviour. PBC would include the perceived influence of both internal (e.g. self-efficacy, skills) and external (e.g. opportunities, constraints) factors and is assumed to reflect past experience as well as anticipated impediments and obstacles. The TPB predicts that the more favourable are people's attitudes and subjective norms with respect to a behaviour and the greater their PBC, the stronger will be their intention to perform the behaviour [10].

However, condom use is not only determined by these psychosocial factors. The concrete act of using a condom must be preceded by other behaviours, called “preparatory behaviours” [8,11,12]. The three preparatory behaviours relevant to condom use are buying condoms, having them available, and discussing condom use with a sexual partner. Van Empelen and Kok [13] suggested that interventions aimed at promoting condom use should not only focus on condom use itself but should also motivate and encourage buying and carrying condoms [13]. This conclusion underlines the important role of preparatory behaviours and how they affect condom use.

In this study, besides the three preparatory behaviours mentioned above, two condom use behaviours were investigated: the use of a condom by *waria* receiving anal sex (receptive anal sex) and the use of a condom by *waria* performing insertive anal sex. We also considered the behaviour of *waria* who, when receiving anal sex, encourage condom use by their sexual partners.

### Objectives

The first aim of this study was to test whether the psychosocial determinants as defined by the TPB could explain three preparatory behaviours of condom use (getting, carrying and offering condoms) and two condom use behaviours (during receptive and insertive anal sex) among *waria*. For each of these behaviours, specific measures of attitude, subjective norms and PBC were developed. Since the study used a cross-sectional design with structured interviews among *waria*, no prospective data on actual behaviours were gathered. Therefore, the psychosocial determinants were related to two proxies of actual behaviour on the one hand, and on the other hand they were related to the intention and actually engaging in the specific behaviour in the past. The second aim of this study was, where possible, to conduct mediation tests to examine whether attitude and PBC mediate the relationships of subjective norms with intention and behaviour.

## Methods

### Recruitment procedure

In total, 210 *waria* were approached, of whom 209 were included in this study. These comprised almost 16% of the last estimated total of *waria* in Jakarta [14]. Subjects were recruited using a sampling cluster procedure with the five municipalities in Jakarta as groups (Central, North, South, East and West), representing all residential locations of *waria*. We recruited participants in the Centre of Jakarta (*n*=23), East Jakarta (*n*=75), West Jakarta (*n*=45), South Jakarta (*n*=45) and North Jakarta (*n*=22) between September and October 2007. One participant from East Jakarta was excluded from this study due to a serious language barrier.

*Waria* often live in small groups, coordinated by a ringleader or *mami*. A *mami* is usually a senior *waria* who provides support for the 5–15 *waria* in her group, including violence protection and promotion of condom use, uptake of HIV testing and regular STI check-ups [15]. The Srikandi Sejati Foundation, the *waria* organization in Jakarta, provided a list of *waria* living in all five districts, and the *mamis* were asked to approach those present and available for inclusion in our study at the time of data collection.

### Interview procedure

The present study was part of a broader study covering a range of behaviours related to condom use and HIV-related health-seeking behaviours. It was approved by the Ethical Committee Psychology at the University of Groningen, the Netherlands. This article only provides data on three preparatory and two condom use behaviours. The interviews were structured and conducted face-to-face. The interview first assessed demographic variables, sexual history and sexual practices. It then assessed five behaviours relevant to condom use, asking the participants about their preparatory behaviours (getting, carrying and offering condoms) and condom use behaviours (during receptive and insertive anal sex). The interviews took 45–60 minutes.

Five interviewers, including the first author, were involved in the data collection. Informed consent was prepared at two levels. First, permission was granted by the *mamis* as the coordinators and leaders of the *waria* in each district. This permission was given after a meeting with all *mamis* to explain the purpose of the study and the possibility of refusing participation. Second, the interviewers introduced themselves to the respondents and informed them about the purpose of the study. Respondents were told that participation was voluntary and that they could withdraw at any time without citing a reason. The respondents were then asked whether they had understood the information and were willing to participate. The actual interviews were conducted after individuals had given verbal consent. For respondents under 18 years of age, verbal consent was given by the *mami* as the person responsible for each group. Unwritten, informed consent was chosen for this study to assure the anonymity and confidentiality of the respondents. Interviews were conducted at different venues; using the networks of the *mamis*, we found quiet places or separate rooms to conduct the interviews. Most of the interviews were conducted in *waria* bedrooms, some in the salons and the rest in the Primary Health Centre or in *mami*s’ living rooms.

## Questionnaire

### Background characteristics

Socio-demographics included age, educational level and marital status. Sexual behaviour was assessed by means of several questions (not presented). The central part of the questionnaire was based on the application of the TPB to preparatory behaviours and condom use by *waria* during anal sex. The primary focus was on the three preparatory behaviours of getting, carrying and offering condoms, and the two condom use behaviours (i.e. condom use during receptive or insertive anal sex). With regard to all five behaviours, we assessed attitudes, social norms, PBC, behavioural intentions and actual (past) behaviour.

### Attitudes

Attitudes were assessed by means of a 7-point semantic differential scale (inconvenient-fun; harmful-beneficial; boring-exciting; something that can be overlooked-a must; foolish-wise). The items for each behaviour included the following: “Getting condoms is …”; “Carrying condoms when I am going to have sex is …”; “Offering a condom when we are going to have sex is …”; “Always using a condom when I have receptive anal sex is …”; “Always using a condom when I have insertive anal sex is ….” Concerning each behaviour separately, the average item score was computed as the scale score: the higher the score, the more positive the attitude towards the specific behaviour. The Cronbach's alpha of the five scales ranged from 0.84 to 0.90.

### Subjective norms

The format of the subjective norms measurement with regard to all five behaviours was “According to the following people, I should or should not always [engage in the specific behaviour].” The short list of people included the regular partner, regular clients, non-regular clients, friends, “mama-san” and outreach workers. Scales ranged from “should not” (1) to “should” (7). For each separate behaviour, the average item score was computed as the scale score: the higher the score, the more positive the subjective norms regarding the specific behaviour. The Cronbach's alpha of the five scales ranged from 0.81 to 0.86.

### Perceived behavioural control

PBC was assessed using three items. One item assessed the perceived difficulty of each behaviour by means of a 7-point scale: (1) very difficult, difficult, somewhat difficult, not easy, somewhat easy, easy and (7) very easy. An example of an item is: “In the next month, will it be easy or difficult for you to get condoms?” The second and third items assessed how certain it was that respondents would be able to engage in the behaviour. Examples are: “In the next month, how sure are you that you can offer a condom whenever you are going to have sex?” and “In the next month, if I want to, I am sure that I will always be able to use a condom when I have insertive anal sex.” Concerning each behaviour separately, the score of the average item was computed as the scale score: the higher the score, the easier it was perceived to be to engage in the specific behaviour. The Cronbach's alpha of the five scales ranged from 0.72 to 0.88.

### Behavioural intentions

Behavioural intentions were assessed as follows: “In the next month, do you intend to [engage in the specific behaviour]?” Answer categories ranged from (1) never, to seldom, sometimes, regularly, often, very often and (7) always.

### Past behaviour

The five behaviours were assessed with the following items: “In the past month, … did you try to get condoms?”; “… did you carry condoms when you were going to have sex?”; “… did you offer a condom when you were going to have sex?”; “… did your clients/partners use a condom when you had receptive anal sex?”; “… did you use a condom when you had insertive anal sex?” Answer categories ranged from (1) never, to seldom, sometimes, regularly, often, very often and (7) always.

### Data analysis

The aim was to relate the three TPB variables (independent variables/IVs) to the condom-use-related variables (dependent variables/DVs) using linear regression analyses. No covariates were included in these analyses. Two proxies of the actual behaviours were regressed on the TPB variables: intention to engage in each of the five behaviours, and past frequencies of engaging in each of the five behaviours. The five behaviours were the three preparatory behaviours: getting, carrying and offering condoms, and the two condom use behaviours: condom use during receptive or insertive anal sex. In sum, a total of 10 regression analyses were conducted (five for each proxy). Before conducting the analysis summary, statistics were generated to evaluate the distributions of variables. The residuals of the IVs and DVs were distributed normally, meeting the statistical assumptions. Below we report for each model, first on the explained variance and F-statistics of the total regression model, and second on the *β* and its related *t*-test of each of the three TPB variables in the model. Table 1 summarizes these statistics and presents the means and standard deviations of each TPB variable with regard to specific intentions and past behaviours.

**Table 1:** Regression analyses of preparatory behaviour, behaviour intention and behaviour of condom use during receptive and insertive anal sex

	Intention				Behaviour			
	
Variable	*M*	SD	*β*	*R* ^2^	*M*	SD	*β*	*R* ^2^
Getting condom	6.20	1.40			4.57	2.15		
Attitude towards getting condom	31.19	4.88	0.37***	0.30	31.26	4.84	0.37***	0.21
Subjective norms regarding getting condom	27.80	6.09	0.22**		27.95	5.93	0.21**	
PBC regarding getting condom	17.35	3.27	0.15*		17.34	3.29	−0.03	
Carrying condom	12.97	2.08			6.11	1.63		
Attitude towards carrying condom	32.94	3.56	0.48***	0.52	32.94	3.56	0.24*	0.28
Subjective norms regarding carrying condom	28.56	6.44	0.01		28.56	6.44	0.13	
PBC regarding carrying condom	18.75	2.89	0.34***		18.75	2.89	0.29**	
Offering condom	12.02	2.93			5.43	2.04		
Attitude towards offering condom	31.01	5.76	0.48***	0.51	31.03	5.75	0.40***	0.42
Subjective norms regarding offering condom	28.29	6.01	0.06		28.30	5.99	0.26***	
PBC regarding carrying condom	17.17	3.45	0.30***		17.19	3.45	0.14	
Using condom during receptive anal sex	11.93	2.71			4.88	2.14		
Attitude towards using condom during receptive anal sex	38.52	5.37	0.29***	0.50	38.58	4.74	0.09	0.22
Subjective norms regarding use of condom during receptive anal sex	28.36	6.23	0.08		28.63	5.71	0.21*	
PBC regarding use of condom during receptive anal sex	17.68	3.40	0.46***		17.89	3.01	0.29**	
Using condom during insertive anal sex	12.17	2.88			4.90	2.42		
Attitude towards use of condom during insertive anal sex	38.32	6.27	0.27**	0.57	39.23	4.36	−0.02	0.32
Subjective norms regarding use of condom during insertive anal sex	28.14	6.97	0.08		28.47	6.83	0.12	
PBC regarding use of condom during insertive anal sex	17.91	3.52	0.50***		18.15	3.10	0.52***	

PBC=perceived behavioural control.

The M of the three determinants are the means of the individual item sum scores.

* *p*<0.05

** *p*<0.01

*** *p*<0.001.

To test for mediation effects, we used Preacher and Hayes’ SPSS Macro PROCESS (version 17). Because this is a fairly novel method, it will be presented here in some detail. PROCESS has been described by Preacher and Hayes [16] as a method for testing multiple mediators. This procedure yields unstandardized path coefficients for a multiple mediator model and estimates 95% confidence intervals (CI) of the indirect (=mediated) effects using a bootstrapping sample procedure. Assessing an indirect effect by means of a bootstrapping sample procedure is more reliable than testing the significance of the mediation effects, because the sampling distribution of the indirect effects is normal only for large samples. The mediation analysis used here followed the product of coefficients approach, and thus focused on indirect effects rather than individual paths [16].

The mediate test was used here to examine whether the relations between subjective norms (independent variables, or IV) and intention (dependent variables, or DV) and between subjective norms (IV) and behaviour (DV) were mediated by attitude and PBC. The results below indicate a mediation when the CI of the indirect path by a 95% bias corrected bootstrap (based on 5000 bootstrap samples) does not contain zero.

## Results

### Descriptive analyses

In total, 209 *waria* took part in the study. The participants’ mean age was 30 years (ranging from 15 to 67); 23% had been in primary school, 28% had been in school levels 7 to 9, almost 42% had a higher education level and 5% had studied at a university. Most *waria* were unmarried (89.5%), 5% were married and 5.8% were divorced or separated.

Of the 209 *waria*, 88% currently only had sex with men, 66% reported selling sex and had had clients in the past week. Regarding the question about the type of sex they “most often” engaged in, almost half of the *waria* indicated receptive anal sex (45.5%), 2.9% indicated insertive anal sex, 10.5% indicated both types of anal sex, while 34% indicated they most often gave oral sex. Few *waria* indicated receiving oral sex (4.3%), having vaginal sex (0.5%) or having thigh sex (2.4%).

### Predicting preparatory and condom use behaviours

Below we report for each model, first on the explained variance and F-statistics of the total regression model, and second on the *β* and its related *t*-test of each of three TPB variables. Table 1 summarizes these statistics and presents the means and standard deviations of each of the three TPB variables with regard to specific intentions and past behaviours.

### Getting condoms

#### Intention

The regression model including the three IVs explained 30% of the variance in intention to get condoms, *F* (3, 146)=20.63, *p<*0.001. All three IVs were significantly related to intention: attitude (*β*=0.37, *p*<0.001), subjective norms (*β*=0.22, *p*<0.005) and PBC (*β*=0.15, *p*<0.05).

#### Behaviour

The regression model including the three IVs explained 21% of the variance in behaviour to get condoms, *F* (3, 144)=12.45, *p<*0.001. Among these three IVs, two were significantly related to behaviour: attitude (*β*=0.37, *p*<0.001) and subjective norms (*β*=0.21, *p*<0.01).

### Carrying condoms

#### Intention

The regression model including the three IVs explained 52% of the variance in intention to carry condoms, *F* (3, 145)=53.08, *p<*0.001. Among these three IVs, two were significantly related to intention: attitude (*β*=0.48, *p*<0.001) and PBC (*β*=0.34, *p*<0.001).

#### Behaviour

The regression model including the three IVs explained 28% of the variance in the behaviour of carrying condoms, *F* (3, 145)=19.09, *p<*0.001. Among these three IVs, two were significantly related to behaviour: attitude (*β*=0.24, *p*<0.05) and PBC (*β*=0.29, *p*<0.01).

### Offering a condom

#### Intention

The regression model including the three IVs explained 51% of the variance in intention to offer a condom, *F* (3, 145)=49.89, *p<*0.001. Among these three IVs, two were significantly related to intention: attitude (*β*=0.48, *p*<0.001) and PBC (*β*=0.30, *p*<0.001).

#### Behaviour

The regression model including the three IVs explained 42% of the variance in behaviour of offering a condom, *F* (3, 146)=34.73, *p<*0.001. Among these three IVs, two were significantly related to behaviour: attitude (*β*=0.40, *p*<0.001) and subjective norms (*β*=0.26, *p*<0.001).

### Using condom during receptive anal sex

#### Intention

The regression model including the three IVs explained 50% of the variance in intention to use a condom in receptive anal sex, *F* (3, 128)=42.83, *p<*0.001. Two IVs were significantly related to intention: attitude (*β*=0.29, *p*<0.001) and PBC (*β*=0.46, *p*<0.001).

#### Behaviour

The regression model including the three IVs explained 22% of the variance in the behaviour of using a condom during receptive anal sex, *F* (3, 109)=10.17, *p<*0.001. Among these three IVs, two were significantly related to behaviour: subjective norms (*β*=0.21, *p*<0.05) and PBC (*β*=0.29, *p*<0.01).

### Using a condom during insertive anal sex

#### Intention

The regression model including the three IVs explained 57% of the variance in intention to use a condom during insertive anal sex, *F* (3, 129)=55.81, *p<*0.001. Two IVs were significantly related to intention: attitude (*β*=0.27, *p*<0.01) and PBC (*β*=0.50, *p*<0.001).

#### Behaviour

The regression model including the three IVs explained 32% of the variance in the behaviour of using a condom during insertive anal sex, *F* (3, 58)=9.25, *p<*0.001. Among these three IVs, only PBC was related significantly to behaviour (*β*=0.52; *p*<0.001).

### Mediation testing for subjective norms

The findings of the multivariate analyses showed that subjective norms were not related to four intentions and two behaviours. However, in the univariate analyses, all these relations were significant (correlations ranged from 0.32 to 0.52; *p*<0.05). The findings in the multivariate analyses may have resulted from the relationship of subjective norms with intention and behaviour being mediated by attitude and PBC. Therefore, we used Hayes’ mediate test [17] to examine these relations more closely.

### Testing the mediation of subjective norms related to intention

**Figure 1:**
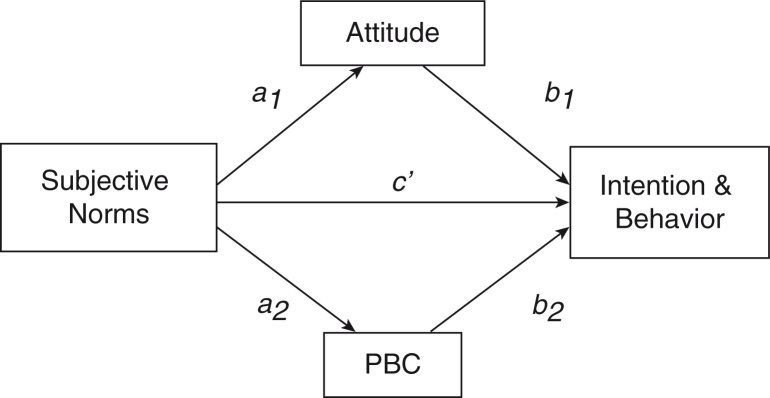
A single-step multiple mediator model.

Simple mediation models (indirect effects) for the intention to carry a condom, the intention to offer a condom, the intention to use a condom during receptive anal sex and the intention to use a condom during insertive anal sex are summarized in Table 2. The indirect effect paths showed that the relationship between subjective norms and intentions with regard to all four behaviours were significantly mediated by attitude as well as by PBC. Since there were more than one mediator, the model is called a single-step multiple mediators model (see Figure 1), in which the total effect of the model (the relationship between subjective norms and intentions is symbolized by *c*) is equal to the direct effect of subjective norms on intention (symbolized by *c1*) plus the sum of the indirect effect of attitude (*a1b1*) and the indirect effect of PBC (*a2b2*). That is, *c*=*c’*+*a1b1*+*a2b2*.

**Table 2:** Indirect relationships of subjective norms with intention and behaviour, mediated by attitude and perceived behavioural control

			Bootstrapping BC 95% CI	
			
	Point estimate	SE (boot)	Lower	Upper
Intention to carry condom				
Attitude	0.0760	0.0257	0.0344	0.1393^a^
PBC	0.0369	0.0198	0.0103	0.0890^a^
Intention to offer condom				
Attitude	0.0765	0.0268	0.0341	0.1403^a^
PBC	0.0683	0.0285	0.0256	0.1415^a^
Intention to use condom during receptive anal sex				
Attitude	0.0581	0.0303	0.0137	0.1312^a^
PBC	0.0975	0.0332	0.437	0.1750^a^
Intention to use condom during insertive anal sex				
Attitude	0.0624	0.0388	0.0104	0.1582^a^
PBC	0.1233	0.0378	0.0558	0.2028^a^
Behaviour to carry condom				
Attitude	0.0294	0.0141	0.0052	0.0612^a^
PBC	0.0247	0.0141	0.0067	0.0635^a^
Behaviour to use condom during insertive anal sex				
Attitude	−0.0044	0.0291	−0.0884	0.0365
PBC	0.0838	0.0416	0.0243	0.2025^a^

PBC=perceived behavioural control.

a Significant mediation.

The total effects of subjective norms on all four intentions were significant: intention to carry a condom: *c*=0.116, *p*<0.001; intention to offer a condom, *c*=0.175, *p*<0.001; intention to use a condom during receptive anal sex, *c*=0.190, *p*<0.001; intention to use a condom during insertive anal sex, *c*=0.218, *p*<0.001.

### Testing the mediation of subjective norms as related to behaviour

Simple mediation models (indirect effects) for the behaviour of carrying condoms and use of condoms during insertive anal sex are summarized in Table 2. The indirect effects showed that the relationships of subjective norms with the behaviour of carrying condoms were significantly mediated by attitude as well as by PBC. With regard to use of a condom during insertive anal sex, the indirect paths showed that only PBC mediated the relationship of subjective norms and behaviour.

The total effect of subjective norms on the behaviour of carrying a condom was significant, *c*=0.086*, p*<0.001, and the total effect of subjective norms on the behaviour of using a condom during insertive anal sex was significant, *c*=0.120, *p*<0.05.

## Discussion

To date, no published data are available on the TPB variables in predicting condom use among *waria*. This cross-sectional study showed the relevance of TPB in explaining preparatory behaviours and condom use by looking at psychosocial factors. Such knowledge of these determinants of behaviour is essential for systematically developing interventions to promote condom use in the target group in order to prevent HIV transmission [18].

In accordance with several previous studies of the TPB on HIV protection behaviours, the TPB variables differed in their predictive power. With regard to intention, attitude and PBC were consistently significant predictors; attitude was the strongest predictor of intention for all three preparatory behaviours, and PBC was the strongest predictor of intention for condom use, either during receptive or insertive anal sex. The explained variance in intention ranged from 30 up to 57% (Table 1). In earlier studies, the TPB variables explained variance in intention of condom use ranging from 17 to 69% [9]. The explained variance in intention in this study was thus in line with earlier studies.

The TPB variables were differentially related to the five behaviours. Attitude was associated with three behaviours, PBC with three behaviours and subjective norms with two behaviours. The explained variance in behaviour ranged between 21 and 42% (Table 1). A review of 26 studies showed that the overall average explained variance in behaviour was 34%, varying from 15.6% (clinical and screening behaviours) to 42.3% (HIV-related behaviours) [5]. Thus, the explained variances of behaviour in this study span within the range of explained variances in earlier studies.

Regarding TPB determinants, and particularly with regard to predicting intentions, it seemed at first that subjective norms were less important. However, mediation tests revealed that the relationship between subjective norms and intentions was completely mediated by attitude and PBC in four of the five behaviours. In two of the five behaviours, the relationship between subjective norms and past behaviour was completely mediated by one or both of the other determinants. Psychologically, these data suggest that subjective norms may contribute to the development of attitudes and PBC.

Prior to concluding the implications of the current study for HIV prevention interventions, several limitations need to be mentioned. First of all, as this study was cross-sectional, we do not predict intention and behaviour. Rather, we do present these DVs in our study as the best predictors of future behaviour [10,19]. Second, a structured face-to-face interview was conducted to roll out the survey. This method was chosen with great care to cover the variance in literacy levels of the participants, which ranged from very low or illiterate to very high. However, the interview format may have resulted in socially desirable answers or interviewer biases [20]. In addition, explanations and different wordings of questions to adjust to the literacy levels of participants may have lowered the standardization of the interview, thereby threatening its internal validity. An attempt was made to minimize these potential drawbacks by providing a structured questionnaire and by training all the interviewers to standardize the question delivery as much as possible.

## Conclusions

Our current results have implications for the development of prevention interventions to promote preparatory and condom use behaviours in the *waria* population. First, all three preparatory behaviours (i.e. getting, carrying and offering condoms) and the two condom use behaviours (i.e. during receptive and insertive anal sex) should be targeted. Previous studies found that preparation, specifically for condom use, is an important prerequisite for safer sex [11,12]. Second, our study suggests that prevention interventions should target all three psychological factors defined in the TPB (i.e. attitude, subjective norms and PBC).

When it comes to attitudes, the present study shows that there is still substantial variance; not all *waria* have positive attitudes towards condom use. Attitudes are formed on the basis of knowledge and beliefs as to the consequences of one's behaviour. *Waria* must, therefore, be continuously educated about the risks of unsafe sex. This can be done by using well-designed and targeted educational materials, including posters and leaflets, but also by asking the *mamis* to warn their *waria*.

Trying to change attitudes may, however, not be sufficient. For example, our study showed that with regard to all five behaviours, PBC is related to intention. We might therefore consider targeting PBC. A person's PBC is influenced by barriers they experience with regard to engaging in a specific behaviour; the removal of such barriers could make positive behaviour easier; this could be facilitated by intervention components. For example, to strengthen a *waria* intention to get condoms, policies and resources could be developed to make condoms available and visible in locations where they are really needed (i.e. locations commonly used for hanging out or commercial sex settings such as parks, railway stations or along the roads). Support of such policies is critical to assure continuity. The PBC of *waria* might also be influenced by teaching them the necessary skills of negotiating condom use and actually putting on a condom. Making these potentially difficult tasks easier could empower *waria* and increase their self-efficacy.

Besides attitudes and PBC, subjective norms should also be targeted. Based on a narrow conceptualization of subjective norms as a psychological factor, interventions might aim to convince *waria* that there is a broad consensus on what people think that they should do. The prevention intervention in itself could not only provide *waria* with condoms but also send the message that the use of condoms is socially desired. Based on a broader conceptualization of subjective norms, interventions might also target the social environment to create a solid supportive network to encourage condom use behaviours; local stakeholders, gate keepers, community and religious leaders would at least need to be positive about condom use as prevention for HIV.

To conclude, face-to-face interviews with over 200 *waria* provided unique data on the psychology behind their condom use. These data form a valuable background for the development of effective preventive interventions to control the spread of HIV transmission among *waria* and to promote their health.
